# Astroglial Integrins in the Development and Regulation of Neurovascular Units

**DOI:** 10.1155/2012/964652

**Published:** 2012-12-10

**Authors:** Hironobu Tanigami, Takayuki Okamoto, Yuichi Yasue, Motomu Shimaoka

**Affiliations:** ^1^Department of Anesthesiology, Osaka Medical Center for Cancer and Cardiovascular Diseases, 1-3-3 Nakamichi, Higashinari-ku, Osaka 537-8511, Japan; ^2^Department of Molecular Pathobiology and Cell Adhesion Biology, Graduate School of Medicine, Mie University, 2-174 Edobashi, Mie, Tsu City, Japan

## Abstract

In the neurovascular units of the central nervous system, astrocytes form extensive networks that physically and functionally connect the neuronal synapses and the cerebral vascular vessels. This astrocytic network is thought to be critically important for coupling neuronal signaling activity and energy demand with cerebral vascular tone and blood flow. To establish and maintain this elaborate network, astrocytes must precisely calibrate their perisynaptic and perivascular processes in order to sense and regulate neuronal and vascular activities, respectively. Integrins, a prominent family of cell-adhesion molecules that support astrocytic migration in the brain during developmental and normal adult stages, have been implicated in regulating the integrity of the blood brain barrier and the tripartite synapse to facilitate the formation of a functionally integrated neurovascular unit. This paper describes the significant roles that integrins and connexins play not only in regulating astrocyte migration during the developmental and adult stages of the neurovascular unit, but also in general health and in such diseases as hepatic encephalopathy.

## 1. Introduction

**Figure 1:**
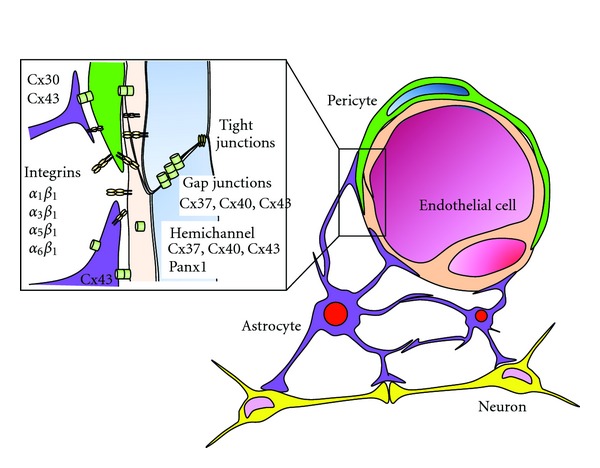
Astrocytic networks are essential to neurovascular units. Astrocytes extend the perivascular processes onto cerebral vascular endothelial cells and pericytes, thereby enwrapping the BBB blood vessels [1, 2]. These astrocytic perivascular processes express integrins that guide and stabilize the endfeet attachments and connexins that support long-range communication in the astrocytic network. Pericytes and endothelial cells express integrins and connexins [3]. Astrocytes extend the perisynaptic processes to the neuronal synapses, thereby surrounding the neuronal synaptic gap, forming a “tripartite synapse” therein [4, 5].

The microenvironment of the central nervous system (CNS) must function correctly if the signaling activities of neurons are to be fully supported [1, 2, 6]. The blood-brain barrier (BBB) represents the key to maintaining an optimal brain microenvironment, which it is accomplished by restricting molecular passages to the brain from the systemic circulation. The major cellular components that constitute the BBB are cerebral microvessel endothelial cells (CMECs), astrocytes, and pericytes (Figure 1). The ability of the BBB to physically separate the brain microenvironment from its systemic counterpart principally derives from the tight intercellular junctional complexes that form among BBB endothelial cells. This mechanism blocks the paracellular passage of ionic molecules, thereby restricting molecular passage solely through transcellular routes. While allowing for the diffusion of gaseous and small lipophilic molecules through the lipid membrane, these transcellular routes utilize specific transporters that mediate the regulated trafficking of selective hydrophilic molecules at the BBB. In this way, large hydrophilic molecules such as peptides and proteins are usually unable to penetrate the BBB and are thereby excluded from the CNS microenvironment.

Astrocytes make extensive contact not only with neurons, but also with BBB vascular endothelial cells and pericytes (Figure 1) [7]. Throughout the brain vasculature, astrocytes, neurons, and endothelial cells are interconnected and thus form a closely knitted coupling cellular network known as the neurovascular unit [8]. Astrocytes were once thought to play merely a housekeeping role in regards to neurons by providing them with structural and nutritional support. Today, increasing evidence has shown that astrocytes play a pivotal role in the development and regulation of the BBB, as well as in neuronal synapses, thereby making critical contributions to the maintenance of CNS homeostasis [1, 2, 6].

Astrocytes have been reported to be aberrantly activated in several pathologies including chronic pain [9]. In addition, the BBB has been shown to become leaky in response to peripheral inflammatory pain [10, 11]. However, few studies have addressed, thus far, a potential role by which the molecular and cellular interactions at the neurovascular unit might play in the pathogenesis and progression of chronic pain. Addressing this problem could lead to innovative therapies that ameliorate chronic inflammatory pain [12]. Therefore, we believe that it is of great interest for pain researchers to read recent progress made in understanding the roles that astrocytes play in their interaction with both BBB endothelial cells and neurons. Here we review the roles of astroglial cell adhesion molecules in the neurovascular unit in the context of not only overall health, but also diseases such as hepatic encephalopathy. Our emphasis will be on integrins, the foremost family of cell-adhesion molecules, whose mediation of cell migration and astrocyte adhesion [13, 14] ideally positions them for further interaction with BBB endothelial cells, as well as with neurons in the neurovascular units.

## 2. The Role of the Astrocytic Network Vis-à-Vis Neurovascular Units

Astrocytes extend endfeet, which consist of perivascular processes, onto the intraparenchymal regions of cerebral vascular endothelial cells and pericytes, thereby enwrapping BBB blood vessels (Figure 1) [1, 2]. These astrocytic perivascular processes express several receptors and channels (e.g., potassium channels, aquaporin 4, and glucose transporters) at the luminal surface, which are thought to be important for regulating BBB endothelial functions [6].

Astrocytes extend other types of processes to the neuronal synapses, thus facilitating physical contact with both pre- and postsynaptic neuronal cell membranes (Figure 1) [1, 2]. These astrocytic perisynaptic processes surround the neuronal synaptic gap, forming a “tripartite synapse” therein [4, 5]. At the tripartite synapse, astrocytes sense neuronal signaling activities (e.g., concentrations of neurotransmitters) and respond to them by secreting gliotransmitters [15, 16]. A notable example of the latter is the amino acid D-serine, which is produced exclusively in astrocytes [17]. D-serine binds to an agonist (glycine)-binding site of the NMDA receptor, thereby activating the opening of the Ca^2+^ channels. The D-serine secreted from astrocytes into the synapse has been shown to contribute to long-term potentiation [18]. 

In this way, astrocytes construct a physical network that connects the neuronal synapses with the cerebral vessel cells, thereby compartmentalizing neurons and cerebral vessels within the neurovascular units. In the context of neurovascular units, astrocytes are thought to play a central role in coupling neuronal signaling and metabolic activities with the cerebral blood flow. In order to establish this elaborate astrocytic network, which enables robust neurovascular coupling, the spatiotemporal positioning of astrocytes and their processes must be carefully calibrated and fine-tuned. Integrins, which govern the migration of various cell types under a wide-range of different microenvironments, have emerged as a key player in the development of neurovascular units [8]. Conditional knockout of either alphaV [19] or beta8 integrin [20] in the CNS has been shown to compromise proper development of neurovascular units, resulting in premature death due to multiple brain pathologies including cerebral hemorrhage. Interestingly, mice deficient in a cytokine TGF-beta have been shown to develop CNS pathologies that are nearly identical to those in mice lacking alphaV or beta8 integrin [21]. The prematurely developed neurovascular units observed in TGF-beta, as well as in alphaV and beta integrin, knockout mice can be explained by the fact that alphaVbeta8 and alphaVbeta6 integrins possess a unique ability to activate the TGF-beta-signaling pathway [22]. TGF-beta is stored in the extracellular matrix in a biologically latent inactive form, in which TGF-beta is caged in latency-associated peptides. The binding of alphaVbeta8 and alphaVbeta6 integrins to the RGD motif in latency-associated proteins leads to conformational changes in the cage structure. Subsequently, in cooperation with mechanical forces imposed on the extracellular matrices, a biologically active form of TGF-beta is released. 

## 3. Integrins in the Immune and Central Nervous Systems

The following section will briefly descibe the structure and functions of integrins in the central nervous and immune systems. We begin with integrins in the immune system, since their structure and functions have been extensively studied in immune cells [23–25], which share many important features with astrocytes. In addition, recent investigations have revealed that the fundamental machinery that governs integrin functions is well conserved across various integrin types [26, 27]. Thus, we believe that what has been learned about the integrins in the immune systems will facilitate our understanding of the roles played by integrins in astrocytes.

### 3.1. Integrins in the Immune System

**Figure 2:**
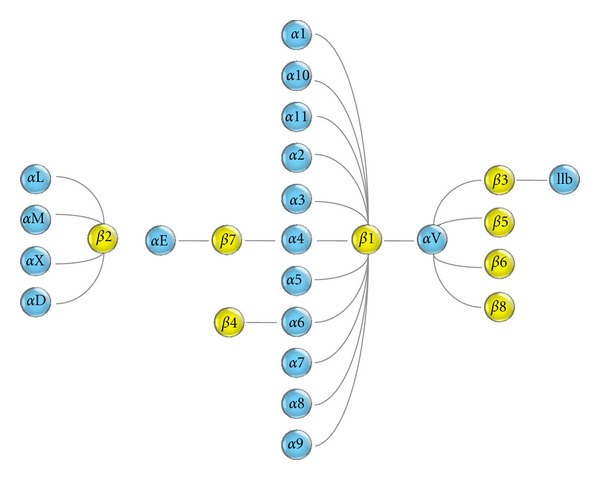
Integrin subunits. Eighteen alpha- and eight beta-subunits form 24 integrin heterodimers.

Integrins represent the largest family of cell-adhesion molecules comprised of noncovalently-associated alpha and beta subunits [23, 26, 28, 29]. Integrins govern cell-to-cell and cell-to-extracellular matrix interactions over a wide range of important biological phenomena. Every cell in the body usually expresses more than one kind of integrins. Not only do integrins support the shear force-resistant stable firm adhesion of immune cells, they are also involved in the dynamic adhesive interactions observed in the cellular polarization and cell migration of endothelial, mesenchymal, and epithelial cells. Integrin-dependent physiological processes include tissue morphogenesis, inflammation, wound healing, and the regulation of cell growth and differentiation. In vertebrates, 18 different integrin alpha subunits and eight different integrin beta subunits have been described thus far, forming at least 25 alpha/beta heterodimeric integrin receptors (Figure 2). Integrins are probably the most structurally and functionally intricate family of cell-adhesion molecules yet known. Astroglial cells have been shown to express multiple beta1, beta2, beta3, and beta8 integrins [13]. 

The ability of integrins to transmit bidirectional signals across the plasma membrane makes this family of cell-adhesion molecules unique [26, 27]. The activation of intracellular-signaling pathways, as triggered by other receptors (e.g., receptors coupled to G proteins or tyrosine kinases), eventually reaches, and impinges on, integrin cytoplasmic domains. This heightens the activity of the extracellular headpiece towards ligand binding via global conformational changes (inside-out signaling). Conversely, when ligand binds to the extracellular part of the integrin, it initiates intracellular signaling via conformational changes to the cytoplasmic domains (outside-in signaling). The dynamic conversion of integrin conformations between nonadhesive and adhesive states is made possible by such bidirectional signaling.

The cell adhesion and migration mediated by integrins are vital to the abilities of immune cells to position themselves at the right place and time for mounting immune responses to fight infections [24]. Integrins on immune cells play a critical role in their adhesive interactions with endothelial cells during migration to lymphoid organs and extravasation to sites of inflammation. One can better understand the physiologic importance of integrins on immune cells by examining genetic disorders such as leukocyte adhesion deficiency type I (LAD-I) and type III (LAD-III). 

LAD-I is caused by mutations that lose, or severely reduce, the expression of all beta2 integrin heterodimers on the cell surface of leukocytes [30, 31]. Recurrent and often life-threatening bacterial infections and impaired wound healing are the prominent clinical manifestations observed in LAD-I patients, since beta2 integrins are important for host defenses against microorganisms. In LAD-I patients, the capacity of LAD-I neutrophils to adhere to endothelial cells and to migrate to sites of inflammation becomes markedly reduced, in the same way that the ability of LAD-I lymphocytes to undergo antigen- and mitogen-induced proliferation, antibody-dependent killing, and T-cell-dependent antibody production becomes impaired. 

LAD-III is caused by mutations that lose the expression of kindlin-3, an intracellular-signaling protein that activates integrins by binding to integrin beta cytoplasmic tails. In LAD-III patients, while the cell-surface expression levels of integrins are unchanged, the ability of integrins to upregulate their ligand binding activity in response to chemokines and/or the other chemoattractants that activate GPCR signaling is impaired not only in immune cells, but also in platelets [32]. Such integrin dysfunction in platelets can be observed in Glanzmann thrombasthenia, where one finds a lack of integrin alphaIIb/beta3 expression and/or functionality. Kindlin-mediated integrin activation is thought to be conserved across many integrin members including immune cells and platelets.

### 3.2. Structures of Integrin Heterodimers and Integrin Domains

**Figure 3:**
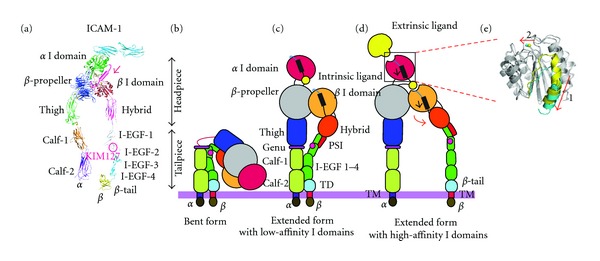
Integrin structures and domains and conformational changes. (a) Integrin extracellular segment model. (b)–(e) Global conformational changes between the bent (b), intermediate (c), and extended (d) conformations. Blowups (e) showing the structures of the high- and low-affinity conformations of the alpha I domain. A piston-like downward shift of the C-terminal helix (arrow 1) is allosterically linked to the conversion of the MIDAS to the high-affinity configuration (arrow 2). Superposition of the high- (blue) and closed low- (yellow) affinity I domain is shown. Regions undergoing significant conformational changes are shaded in color, whereas regions not undergoing such changes are shaded in gray.

Integrin alpha and beta subunits are type I transmembrane proteins composed of a large extracellular segment, a single transmembrane segment, and a short cytoplasmic tail (except for the beta4 subunit, which contains a long cytoplasmic tail). The C-termini of the alpha and beta subunits associate with each other to form a globular ligand-binding headpiece. This headpiece is connected to the plasma membrane via the leg pieces (Figure 3(a)). This complex multidomain organization is a prominent characteristic feature of integrins.

Probably the most important integrin domain is a von Willebrand factor-type A domain of *∼*200 amino acids known as an inserted (I) domain that is included in half of the alpha subunits and all of the beta subunits [23, 33]. These I domains are assumed to be a Rossmann-like fold that contains a metal ion-dependent adhesion site (MIDAS) located on the top, whereas its C- and N-terminal connections are located on the distal bottom face (Figures 3(a) and 3(e)) [23, 34–36]. The ligand-binding affinity of the I domain is regulated by conformational changes. Affinity is dramatically enhanced by a “piston-like” downward axial displacement of its C-terminal helix (arrow 1 in Figure 3(e)). This C-terminal downward shift occurs in tandem with the conversion of the MIDAS to a high-affinity configuration, which binds tightly to ligand (arrow 2 in Figure 3(e)) [37–40]. Conversely, upon binding to ligand, the MIDAS assumes its high-affinity configuration, an action that has been linked to the downward axial displacement of the C-terminal helix [34, 39, 41]. 

Upon the activation of chemokine receptors and several different growth factor receptors, an intracellular signaling cascade is initiated that eventually impinges upon the integrin cytoplasmic tails. Binding to the integrin cytoplasmic domains of the signaling molecules (e.g., talin and kindlin) elicits a response: the integrin cytoplasmic tails begin to separate [42–44]. Dissociation of the alpha and beta subunit cytoplasmic tails is conformationally coupled to a separation of the transmembrane domains from the membrane-proximal segments of the extracellular domains, thereby inducing a switchblade-like opening that leads to an extended conformation [26, 29]. While extending, the outward movement of the hybrid domain is effected, an action which is coupled to the downward piston-like movement of the C-terminal helix of the I domain (Figures 3(b)–3(d)). The downward movement of the beta I domain's C-terminal alpha helix triggers the conversion of the MIDAS into an open configuration, thereby binding to the C-terminal portion of the alpha I domain as an “internal” ligand [45]. This interdomain interaction leads to a downward shift of the alpha I domain C-terminal helix, converting it to the high-affinity conformation, which binds to an “external” ligand.

### 3.3. Integrins in the Central Nervous System 

#### 3.3.1. Formation of the Neuronal Synapse

As described in the Introduction, integrins—while regulating the astrocyte-cerebral endothelial cell interactions that occur in the neurovascular unit—also play a role in mediating neuron-to-neuron, as well as astroglia-to-neuron, interactions in the CNS [13, 16]. At the neuron-to-neuron interaction at synapses, integrins have been shown to modulate synaptic plasticity [46]. By using integrin-inhibiting RGD peptides applied to hippocampus slices, the inhibition of integrins has been shown to suppress LTP [46, 47]. Of note, in one study a fruit fly Drosophila mutant containing a short-term memory defect, now known as the locus of Volado (a Chilean word for “forgetful”), was discovered to encode an alpha integrin [48]. Integrins not only strengthen the physical contact of neurons at the synapse, but are also involved in the signaling that occurs at the interface. A series of investigations using knockout mice that lacked the expression of specific integrins have confirmed and substantiated the roles played by synaptic integrins in the modulation of synaptic plasticity [49–54].

Integrins are thought to be an important player in mediating astroglia-neuron interactions at the tripartite synapse [16]. Astrocytes secrete to the synapses extracellular matrix protein thrombospondins, which interact not only with the alpha2/delta1-1 subunit of the voltage-dependent Ca^2+^ channel on neurons, but also with the integrins on astrocytes and neurons. The former interaction transmits a critical signal for synaptic development [55], while the latter potentially supports the adhesion of glial cells with neurons. It has been demonstrated that neurons are capable of forming, but not receiving, synapses unless physical contact with astrocytes has been established [56]. Integrin alphaVbeta3 on astrocytes has been shown to bind to a glycoprotein Thy-1 abundantly expressed on neurons [57]. Upon binding to neuronal Thy-1, astrocytic integrins emit a signal that activates astrocytes. In turn, upon binding to astrocytic integrins, neuronal Thy-1 receptors undergo clustering, and similarly emit a signal to inhibit neurite outgrowth and to retract neuronal processes [58]. This bidirectional astrocyte-neuron communication via integrins might play an important role in axon repair following neuronal damage. Another investigation has shown that the beta1 integrin on neurons mediates their contact with local astrocytes, thereby facilitating the transmission of the signals that induce neuronal PKC activation, which globally enhances excitatory synaptogenesis [59].

#### 3.3.2. Glial Migration to Sites of Injury and Regeneration

Astrocytes become activated and accumulate at sites of injury in response to a CNS injury, which leads to a release of inflammatory mediators such as ATP, cytokines, and chemokines [9]. This phenomenon is termed reactive astrocytosis. Astrocytosis as well as microgliosis has been implicated in the pathogenesis of multiple chronic pain models [60] and involves a process by which beta1 integrins help regulate astrocyte migration [61]. Beta1 integrins colocalize with matrix metalloprotease-2 at the leading edge of migrating astrocytes. While matrix metalloprotease-2 cleaves and breaks down the mesh of extracellular matrix proteins, integrins simultaneously support cell migration, thereby driving forward movements within tissues.

In spinal cord injury, damaged neurons secrete the chemokine MCP-1, thereby attracting and activating astrocytes [62]. Beta1 integrins have also been implicated in astrocyte migration to sites of spinal cord injury [63]. Astrocytes that migrated to, and were activated at, the dorsal root of the spinal cord released IL-1 and TNF, which have been implicated in the pathogenesis of neuropathic pain [64, 65]. 

The peripheral nervous system contains a subset of glial cells termed Schwann cells. Schwann cells migrate to injured neurons to affect tissue repair, specifically remyelination in injured demyelinated nerves. While integrins mediate Schwann cell migration, the aggrecan produced by astrocytes inhibits it [66]. In ensheathing and myelinating neuronal axons, Schwann cells form a laiminin-deposited organized basal lamina, in which beta1 and beta4 integrins interact with laminin. The Schwann cell-specific conditional knockout of beta1 integrins demonstrated that the formation of normal processes around axons was compromised [67]. Subsequently, Schwann cell-specific knockout of beta4 integrin has shown that the alpha6beta4 integrin plays the preeminent role in axonal regeneration and subsequent myelination [68].

#### 3.3.3. Hedgehog Pathway

The glycoprotein Sonic hedgehog (Shh), an important ligand for the hedgehog-signaling pathway, plays a key role in vertebrate organogenesis [69]. Shh has also been implicated in vertebrate neuronal development [70]. Shh modulates integrin activity and thereby dictates neuronal epithelial cell adhesion [71]. Shh-regulated integrin activity in neural progenitors has been implicated in the coordination of neural tube morphogenesis. 

Astrocytes are an important source of Shh secretion in the BBB microenvironment [72]. During the developmental stages of the neurovascular unit, Shh released from astrocytes binds to Hedgehog receptors expressed on BBB-endothelial cells. This astrocytic signaling to BBB endothelial cells via Shh promotes the maturation of the BBB, thereby maintaining the integrity of the barrier function. Shh-regulated integrin activity might be involved in BBB-endothelial migration and attachment. In addition, at the adult stage, the Shh released from astrocytes suppresses ICAM-1 expression in endothelial cells, thereby functioning to dampen endothelial proinflammatory responses such as occurs in multiple sclerosis. 

## 4. Connexins and Pannexins in Neurovascular Unit

While astrocytic integrins are thought to be important for both guiding and stabilizing the endfeet attachment that enwraps cerebral blood vessels, another family of cell-adhesion molecules known as connexins is enriched at the interface between astroglial and endothelial cells, where they form numerous gap junctions [73]. Gap junctions are built by two hemichannels, which collectively are known as a connexon, which contains six connexin proteins [74]. Gap junctions connect the cytoplasm of neighboring cells and allow the passage of intracellular mediators such as ions, amino acids, small metabolites, and second messengers. In addition, they mediate direct intercellular communication involving electrical coupling and biochemical coupling and play a role in neuroglial interaction and neurotransmission. Hemichannels are also formed by connexin or pannexin proteins, which similarly open on the cell surface, thereby allowing the exchange of small molecules between the cytoplasm and the extracellular environment [75]. Pannexins have a similar protein structure and form a single-membrane channel [76]. Thus, gap junction- and hemichannel-mediated intercellular communication is thought to play an important role in signal transmission in neurovascular units [77, 78]. 

To date, 20 connexins and 3 pannexins have been described in vertebrates. Connexin 43 (Cx43) and connexin 30 (Cx30), which transport ions, glucose, and metabolites intercellularly [79], are highly expressed in astrocytic perivascular endfeet and blood vessel walls in mice [78]. The functions of these connexins have been extensively studied *in vivo* using knockout mice. Cx30-knockout mice were found to possess an altered neurochemical microenvironment characterized by elevated choline levels in the ventral striatum, which potentially caused aberrant behaviors (e.g., heightened emotionality) [80]. Astrocyte-specific Cx43 conditional knockout mice exhibited *∼*50% reduction of intercellular communication at the hippocampus, which is associated with electrophysiological and behavioral abnormalities (i.e., advancing depression and aggravating locomotory activity) [81]. Of note, one investigation using mice with an astrocyte-targeted deletion of Cx43 and a global loss of Cx30 showed that the simultaneous deletion of Cx30 and Cx43 in astrocytes led to the generation of a dysmyelinating phenotype in the hippocampus [82]. Another investigation using mutant mice lacking Cx30 and Cx43 in glial fibrillary acidic protein (GFAP)-positive cells (which included astrocytes) demonstrated that the deletion of Cx43 and Cx30 weakens the BBB [83]. These results show that the astrocytic connexins Cx43 and Cx30 regulate the integrity of the BBB, as well as the homeostasis of the neurovascular units.

Unlike connexins, which either form gap junctional channels or act as hemichannels, pannexins function only as hemichannels. While intercellular molecular transport via gap junctions represents one mode of long-range communication in an astrocytic network, pannexin hemichannels represent another mode of communication. ATP is released through pannexin 1 in astrocytes and neurons, thereby inducing excitation of purinergic receptors such as the P2X7 receptor in neighboring cells [84, 85]. Aberrant pannexin1 function has been linked to the activation of inflammasome [86], neuronal death [87], epilepsy [84], and ischemic brain injury [88].

## 5. Astrocytopathy in Hepatic Encephalopathy

Astrocytopathy, the pathologic involvement of aberrantly regulated astrocytes, has been observed in various diseases [89, 90]. While many of these are secondary, a few important diseases are regarded as primary astrocytopathies including hepatic encephalopathy, in which Alzheimer's type II astrocytosis (enlarged astrocytes with large pale nuclei and prominent nucleoli) have been observed [91]. Hepatic encephalopathy is defined as a disturbance in central nervous system function due to an acute and chronic hepatic insufficiency. Distinctive clinical manifestations include the onset of confusional states that sometimes evolve into a coma [92].

Ammonia plays the central role in the pathogenesis of hepatic encephalopathy [92, 93]. In patients with acute liver failure, hyperammonemia is an important abnormal laboratory finding that often correlates with the severity of the encephalopathy. By contrast, blood ammonia levels in chronic liver failure patient did not necessarily correlate either with clinical severities (e.g., consciousness) [94], or with pathohistological findings (e.g., cerebral edema) of hepatic encephalopathy [95]. This might suggest the emergence, in chronic hyperammonemia, of secondary/alternative mediators and mechanisms that could confound the pathogenic action of ammonia.

As a small molecule of ammonia can pass the BBB and enter the brain, systemic hyperammonemia inevitably leads to increased ammonia levels in the cerebral microenvironment, an exposure that primarily affects astrocytes [96]. This is because the enzyme glutamine synthetase is mainly located in astrocytes within the brain. Astrocytic perivascular processes are strategically positioned at the frontline of the interface with the systemic circulation, where the efficient metabolizing and detoxifying of ammonia occur. Once entering the cytoplasm of astrocytes, ammonia is incorporated into the glutamine synthetase-mediated catalytic conversion of glutamate to glutamine. However, if elevated plasma ammonia levels persisted, it would result in an excessive accumulation of glutamine in astrocytes, thereby causing osmotic swelling of the cells that would ultimately lead to brain edema and increased intracranial pressure. Alternatively, elevated intracellular glutamine levels compromise mitochondrial membrane potential and promote free radical production, thereby disturbing cell-volume regulation in astrocytes [97]. In addition, the swelling of astrocytes impairs their ability to maintain the homeostasis of the neuronal microenvironment (i.e., to regulate extracellular potassium and neurotransmitter concentrations and to dampen oxidative stresses). 

Whereas the involvement of integrins and connexins in the pathogenesis of primary astrocytopathy warrants further investigation, a few reports suggest that these classes of cell-adhesion molecules play important roles in this context. Cx43 has been shown to upregulate in swollen astrocytes during hyperammonemia [98]. This upregulation might serve as a compensatory response for removing extracellular potassium ions. In response to tissue damage, astrocytic glutamine synthetase is downregulated, a process that has been shown to reduce beta1 integrin expression on astrocytes, thereby enhancing cell migration while at the same time suppressing cell adhesion [99]. Thus, glutamine synthetase might play an important role in regulating astrocyte migration during reactive astrocytosis/gliosis and tissue remodeling of the injured brain.

## 6. Concluding Remarks

Several studies in recent years have demonstrated that astrocytes play important regulatory roles in maintaining the proper functionality not only of cerebral vascular endothelial cells at the BBB, but also of neurons at the tripartite synapse. The interaction of integrins with CNS extracellular matrix proteins helps guide astrocytes and their attendant mechanisms to positions ideally suited for maintaining the integrity of the BBB and the synapse during both developmental and adult stages. Integrins and connexins are among the most important molecules regulating astrocytic networks, an infrastructure that couples neuronal signaling and metabolic activity to the cerebral vascular tones, thereby balancing oxygen and energy demands with the supply of cerebral blood flow. Astrocytopathy compromises the ability of astrocytes to maintain the homeostasis and integrity of neurovascular units, thereby undermining critical neuronal functions. Future studies should be carried out in order to better understand the roles that integrins and connexins play in the pathogenesis of specific astrocytopathies.
